# Enhancement of quality induced by ultrasonic-assisted stewing improved the nutritional concentration, emulsifying property, and flavor characteristic of the chicken soup

**DOI:** 10.1016/j.fochx.2025.102184

**Published:** 2025-01-15

**Authors:** Ziyan Yue, Qiuyu Yu, Yuchen Qin, Yuchun He, Jiali Liu, Yingchun Zhu

**Affiliations:** College of Food Science and Engineering, Shanxi Agricultural University, Taigu 030801, China

**Keywords:** Chicken soup, Ultrasonic-assisted stewing (UAS), Nutrients, Micro-nano particles (MNPs), Emulsification property, Volatile organic compounds(VOCs)

## Abstract

To investigate the effects of ultrasonic-assisted stewing (UAS) on the quality of chicken soup, the nutritional content, emulsifying properties and flavor characteristic were detected, and the underlying mechanisms were analyzed. The results showed that UAS led to a milky appearance, improved nutrient content and emulsifying properties, attributed to increased rheological properties, absolute Zeta potential, and reduced particle size and surface tension. Laser scanning confocal microscopy revealed that the micro-nano particles in the UAS group were uniformly sized and densely arranged. When treated for 90 min of UAS, the chicken soup achieved the highest emulsibility. Additionally, sensory and electronic tongue evaluations demonstrated that UAS group had superior taste attributes compared to the control group. The relative concentration of marker VOCs in the UAS group was also higher than in the control group. These findings offer scientific and theoretical insights into the impact of UAS on chicken soup quality.

## Highlights

• Ultrasonic-assisted Stewing (UAS) promoted the nutrients in the chicken soup.
• UAS improved the emulsifying properties and stability of chicken soup.
• Micro-nano particles of the UAS samples mere uniform in size and densely arranged.
• The flavor of the chicken soup was enhanced through UAS.

## 1 Introduction

Soup holds an important place in people's diets, and there is a saying, “Better to eat without meat than without soup”. Chicken stands out as a versatile, comprehensive and economical food source, with advantages such as low fat and cholesterol levels, high protein content, and low calories (Cao et al., 2021). Chicken soup is a typical example of traditional Chinese food culture and is known for its delicate flavor and rich nutrition (Wu et al., 2024). As a nutritious dish, it has been welcomed by a wide range of consumers. Chicken soup is abundant in amino acids, minerals, microelements, and other important nutrients that help enhancing bodily strength, boost human metabolism, and prevent disease (Gu & Li, 2020). During the stewing process, nutrients and flavor components such as high-quality proteins, functional peptides, flavor peptides, flavor nucleotides, and free amino acids of chicken are fully extracted into the soup, making it delicious, easy to digest and absorb.

The traditional cooking methods of soup are mainly boiling and stewing, which present problems such as long cooking time and uneven heating, posing challenges for industrial chicken soup food production. To control and improve the quality of soup while accelerating the process, emerging technologies such as ultrasonic and microwave have gradually gained attention (Liu et al., 2023). Ultrasonic technology, a new green non-thermal processing method, features strong penetration, low energy consumption, high efficiency, and good repeatability (Visy et al., 2021). Ultrasonic-assisted stewing (UAS) effectively enhances food processing techniques by utilizing cavitation and thermal effects. UAS has diverse applications in processes such as extraction, emulsification, sterilization, reaction rate studies, and crystallization techniques (Singla & Sit, 2021). Studies have shown that UAS can enhance the nutritional content, emulsifying properties, and aroma profile of soups, as demonstrated in the preparation of halibut bone soup (Zhu et al., 2021) and chicken soup (Qi et al., 2023). Despite these advancements about UAS, the underlying mechanisms of UAS improves the quality in chicken soup remain insufficiently understood.

During the stewing process, molecular interactions and chemical bonds reactions lead to the formation of micro-nano particles (MNPs) through self-assembly (Yao et al., 2021). These MNPs, composed of proteins and lipids, play a critical role in stabilizing oil-in-water emulsions and enhancing the sensory properties of soups (Ke et al., 2017). Flavor, comprising both volatile organic compounds (VOCs) and taste-active components, is a critical determinant of the quality and consumer acceptance of chicken soup. The distinctive aroma of stewed chicken soup originates from its complex chemical matrix, with aldehydes, alcohols, and ketones primarily formed through lipid oxidation, while furfural and furan compounds are generated via Maillard reaction pathways (Wang, Guo, Wen, et al., 2024). Gas chromatography-ion mobility spectrometry (GC-IMS), as an innovative technology, provides fingerprint plots that visually distinguish differences in VOCs within food (Sun et al., 2023). Sensory evaluation and electronic tongue analysis further bridged instrumental data with human perception and quantifying taste attributes such as umami, saltiness, and bitterness. These methods offer valuable insights into the emulsification properties and flavor profile, providing a basis for determining the quality and consumer acceptance of chicken soup.

This study aims to explore the impact of UAS on the nutritional content, emulsification properties, and VOCs concentration of chicken soup. Key parameters such as nutritional composition, rheological properties, emulsification capacity, particle size, Zeta potential, and surface tension were analyzed. Microscopic techniques were used to observe MNPs formation, and GC-IMS was employed to characterize aroma compounds. Additionally, sensory evaluation and electronic tongue analysis were conducted to assess the taste and flavor profile of the chicken soup. Unlike previous studies, this research provides novel insights into the mechanisms of MNPs formation and flavor enhancement induced by UAS and a theoretical basis for the application of UAS technology in the industrial production of high-quality chicken soup.

## 2 Materials and methods

### 2.1 Experiment materials and reagents

The Chinese three-yellow chickens, aged approximately 6 months, were obtained from a local market (Shanxi, China) and quickly delivered to the laboratory wrapped in ice. Subsequently, the chicken carcasses were promptly stored at −2 °C until needed. Reagents and chemicals are sourced from Sinopharm Chemical Reagent Company (Shanghai, China).

### 2.2 Preparation of chicken soup

Chicken carcasses were divided into 3.0 ± 2 cm^3^(50 ± 5 g). Then they were thoroughly blended and cleaned. They were subjected to a pre-cooking process in boiling water for a duration of 1 min, after which they were taken out and subsequently cooled in ice water. The precooked chicken nuggets and purified water were contained within heat-resistant plastic bags made of nylon and polyethylene, which are suitable for high-temperature cooking, with each bag containing 200 g of chicken nuggets and 600 g of water. The experiment utilized an ultrasonic stewpot characterized by an internal volume of 280 mm^3^, equipped with 12 ultrasonic probes, each featuring a tip diameter of 66 mm, positioned at the base of the stewpot (THC-1000SF, Tianhua Ultrasonic Electronic Instrument, Jining, China). For the control group, a plastic bag containing chicken nuggets and water was placed directly into the stewpot without UAS, and the stewing time began when the water temperature in the stewpot reached 95 °C, The total stewing time was 2 h. For the UAS group, the ultrasound time and stewing time also began when the water temperature reached 95 °C, with a total stewing time of 2 h, and the samples underwent UAS during the stewing process. UAS was implemented every half hour in a cycle, consisting of ultrasonic assisted stewing (20.4 kHz, 800 W) for the first 20 min followed by a rest period for the next 10 min, as required by the equipment. The control samples and UAS samples were taken every half hour and labeled as C0, C30, C60, C90, C120, U0, U30, U60, U90, U120, respectively. Subsequent to their removal, the bags were subjected to cooling at room temperature through the application of ice water. The chicken nuggets and other impurities were then separated from the mixture utilizing gauze, yielding samples of chicken soup.

### 2.3 Determination of nutritional components

The nutrient composition analysis was conducted following the established guidelines AOAC (1990). The concentration of soluble solid was determined using the method described by Lai et al. (2022). The protein concentration was measured by Kjeldahl nitrogen determination method with a conversion coefficient of 6.25. Lipid content was assessed using chloroform-methanol extraction procedures. Total sugar content was assessed using the phenol‑sulfuric acid method. The ash content of the sample was estimated by high temperature incineration in a Muffle furnace.

### 2.4 Determination of rheological properties

The rheological properties of the chicken soup were determined using the method described by Yue et al. (2024) at 25 °C with a DHR1 device (TA Instruments Ltd., Crawley, UK). A 40 mm parallel plate configuration with a fixed 1000 μm gap was used. The shear rate was programmed to increase linearly from 0 s^−1^ to 300 s^−1^, during which shear stress and apparent viscosity were recorded throughout the process.

### 2.5 Determination of emulsifying properties

The method described by Yue et al. (2024) was utilized to determined the emulsifying activity and emulsion stability, with only slight adjustments. In each groups, 20 μL of chicken soup were added in equal parts to 4 mL of 0.1 % sodium dodecyl sulfate (SDS) solution, and absorbance readings at 500 nm wavelength were recorded at 0, 10 min.

### 2.6 Determination of particle size and zeta potential

The potentiometer analyzer Nano-ZS90 (Malvern Instruments Ltd., Worcestershire, UK) was utilized to determined the particle size and Zeta potential of the sample (Yue et al., 2024). To be specific, 1 mL of the chicken soup was placed in a cuvette. It is important to prevent gas bubbles from forming and introducing foreign matter into the cuvette in the addition process.

### 2.7 Determination of surface tension

Air-water surface tension was determined using a drop shape analyzer (DSA25, Krüss, Germany) employing the pendant drop technique (Cheng et al., 2023). Specifically, a droplet of the CD aqueous solution, formed from top to bottom, was generated using a precision syringe equipped with a needle of 1.826 mm in diameter. The morphology of this droplet was taken via a high-speed camera. Subsequently, the profile of the droplet was analyzed by applying the Young-Laplace equation to the obtained image.

### 2.8 Observation under digital camera and optical microscope

Images of chicken soup appearance were recorded with digital photography equipment. The chicken soup was examined using a Nikon 80 optical microscope (Nikon Hong Kong Holdings Co., Ltd., Hong Kong, China). To be specific, 2.5 μL of chicken soup was placed in the center of a clear glass slide, then a covering glass was placed to ensure that the droplets are evenly distributed and prevent bubbles from forming. The slide was then placed on a microscope table equipped with a 40× objective lens and a 10× eyepiece for viewing the chicken soup.

### 2.9 Observation under laser scanning confocal microscope

Chicken soup was observed using a laser scanning confocal microscope, employing a method outlined in a study by Wu et al. (2024). Specifically, mix 1 mL of equal portion sample with 20.0 μL of Nile Red (prepared at 0.1 % concentration in anhydrous alcohol) and 20.0 μL of Nile blue (prepared at 0.1 % concentration in ultra-pure water). This mixture was then allowed to sit in the dark for 30 min to ensure that the ingredients of the soup are thoroughly stained. The 2.0 μL staining solution was then placed on the slide and carefully covered. The glass slide was left at room temperature for 20 min, after it was air-dried and subsequently observed. Imaging using DeltaVision OMX SR laser scanning confocal microscopy (General Electric Company, Massachusetts, USA), Equipped with 60×/1.42 NA objective lens. Various fluorescent materials were excited by 488 nm and 633 nm.

### 2.10 Determination of volatile flavor compounds by GC-IMS

The GC-IMS analyzer (GAS, Dortmund, Germany) was utilizing to determine the concentration of VOCs (Wang, Huang, Wang, et al., 2024), equipped with an MXT-WAX capillary column (130 m × 0.53 mm × 1 μm). In a 20 mL headspace bottle fitted with a lid, 7 g ± 0.1 g of chicken soup was added and incubated at 60 °C with shaking at 500 rpm for a duration of 20 min, using a shaker-incubator. Subsequently, 500 μL of headspace bottle was injected into the heated injector at 80 °C. The flow rate was initially set at 2 mL/min for 2 min, gradually increased to 100 mL/min over 18 min, and then maintained at 100 mL/min again for 10 min. VOCs were identified by comparison with drift time and RI values in GC-IMS NEST library. The retention index (RI) was calculated using the external reference of C4-C9 n-ketone. The results were presented in the form of peak intensity.

### 2.11 Determination of electronic tongue

A 25 g sample was diluted to 250 mL and sequentially filtered through 0.45 μm and 0.22 μm filters. Then, 35 mL of the filtered chicken soup was transferred into the sample cup of the electronic tongue and positioned on its automatic injector according to a predetermined sequence. The electronic sensor was first cleaned in a cleaning solution for 90 s, followed by a 120-s cleansing with the first reference solution. Next, the second reference solution was applied for 120 s to clean the sensor. Afterward, the sensor was calibrated to the equilibrium position for 30 s before being transferred to the sample cup for a 30-s testing cycle. After each test, the sensor was sequentially rinsed in two reference solutions for 3 s each. Each sample was tested four times, with the initial dataset discarded. The average of the remaining three datasets was calculated to analyze the taste components.

### 2.12 Determination of sensory evaluation

For sensory evaluation, 60 mL of chicken soup was placed in a clear plastic container and kept in a preheated water bath at 45 °C until served. The samples were assigned random three-digit codes to eliminate bias. The panel included 12 postgraduate (7 females and 5 males) aged 20–26 years. Panelists evaluated color, texture, oily, taste, aroma and overall acceptability on a six-point hedonic scale from 1 (extremely dislike) to 15 (extremely like). The taste evaluation was conducted in the laboratory of the College of Food Science and Engineering at Shanxi Agricultural University (Jinzhong, Shanxi, China). Shanxi Agricultural University does not require ethical permission for sensory research. All participants signed an informed consent before the sensory evaluation. The rights and privacy of all participants were protected during the execution of the study.

### 2.13 Statistical analysis

Three biological replication was performed on all samples. One-way ANOVA was performed using IBM SPSS Statistics 22.0, followed by Tukey's HSD post-hoc test to determine specific group differences. *P* < 0.05 was considered as statistically significant. VOCs were analyzed using the built-in software and plug-ins of the GC-IMS instrument. Principal compound analysis (PCA) and orthogonal partial least squares discriminant analysis (OPLS-DA) were performed using Metaboanalyst 5.0 software. The OPLS-DA model was utilized to calculate variable importance in projection (VIP) values. Origin 2024 software was for visualization and data processing.

## 3 Results and discussion

### 3.1 Effect of UAS on nutritional components of chicken soup

**Fig. 1:**
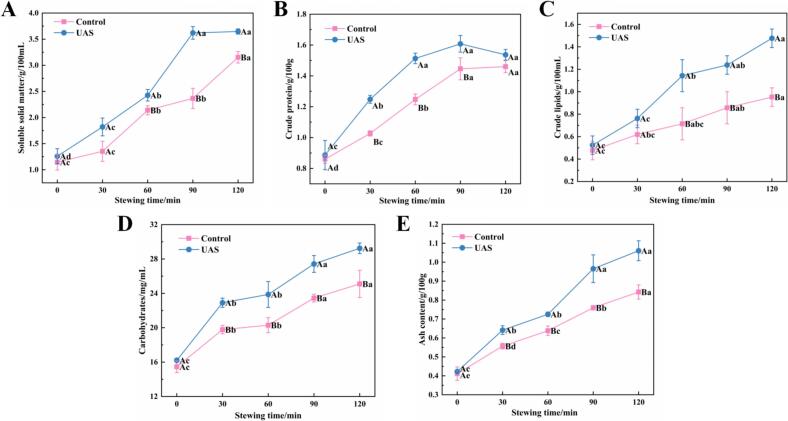
The changes of macromolecular nutrition content of chicken soup of different treatment method: A: the soluble solid content, B: the crude protein content, C: the crude lipid content, D: the carbohydrate content, E: the ash content. Note: Error bars represent standard errors obtained from triplicate sample analysis. Different lowercase letters indicate significant differences (*P* < 0.05) between stewing times, different capital letters indicate significant difference (*P* < 0.05) between the control group and UAS group.

The soluble substance content was identified a crucial marker for evaluating the quality of chicken soup, indicating the total dissolution of nutrients and taste substances (Li, Fan, et al., 2020). The soluble substance content of the two groups in chicken soup rose with extended stewing duration (Fig. 1A). This is because of the rapid dissolution of soluble solids in the initial stages of chicken soup preparation, mainly from easily soluble components found in chicken muscle, including glycogen, minerals, and amino acids. These are subsequently supplemented by soluble compounds released from deeper muscle tissues, and eventually by larger, insoluble macromolecules (Lin et al., 2020). After 120 min of UAS, the soluble solid content reached 3.64 g/100 mL, which was 0.5 g/100 mL more than that in the control group. The shock waves and shear forces generated by the mechanical crushing and cavitation of UAS resulted in an increase in soluble solids. This process could disintegrate the macromolecular nutrients in chicken meat and promote their dissolution (Zhu et al., 2021). Furthermore, when combined with UAS during the stewing process, the soup was subjected to elevated pressure and temperature conditions, which facilitated the migration and distribution of nutrients within the soup.

The richness of proteins, lipids, and sugars not only enhanced the nutrient content of the soup but also imparted a distinctive flavor (Zhu et al., 2021). As the stewing time increased, the content of crude protein (Fig. 1B), crude lipids (Fig. 1C), total sugar (Fig. 1D) and ash (Fig. 1E) in all samples also increased. The main components of crude protein found in chicken soup include chondroitin, collagen, and free amino acids (Zhu et al., 2019), and its content generally showed an increasing trend with the prolongation of stewing time. As stewing time prolonged, the rise in lipid content was attributed to the migration of fat from the chicken's subcutaneous tissue into the soup at high temperatures (Zhang et al., 2021). The hydrolysis of glycosidic bonds resulted in the degradation of glycogen components within the raw meat and the disruption of muscle fiber cells in the process of stewing. This results in the release of intracellular sugar, which increases the total sugar content in chicken soup (Lin et al., 2020). The mineral elements present in chicken, such as K, Na, Ca, Zn, Fe, progressively migrate into the soup throughout the stewing process, the ash concentration in the soup gradually increases as the stewing continues (Qian et al., 2019).

Compared to the control group, UAS facilitated the transfer of nutritional components from chicken meat to the soup. The contents of protein (1.46 g/100 g vs 1.54 g/100 g), crude lipids (0.95 g/100 mL vs 1.48 g/100 mL), total sugars (28.52 mg/mL vs 25.10 mg/mL) and ash (0.84 g/100 g vs 1.06 g/100 g) in the UAS group significantly increased after 120 min of UAS. This was primarily attributed to the elevated pressure, temperature, and shear force generated by the mechanical and cavitation effects of UAS, which enhanced the transfer of large molecular substances from the chicken into the soup. In the UAS group, the contents of soluble solids, crude lipids, total sugar and ash reached higher levels by 90 min of UAS, and peaked at 120 min of UAS, but there were no significant differences between the 90-min and 120-min UAS of chicken soup (*P* > 0.05). However, the chicken soup with 90-min of UAS had the highest crude protein content (1.61 g/100 g). This reduction in crude protein content at 120 min of UAS may have resulted from intensified protein denaturation caused by the combined effects of ultrasound and heat treatment (Qi et al., 2023).

### 3.2 Effect of UAS on emulsifying properties of chicken soup

#### 3.2.1 Rheological properties of chicken soup of different treatment method

Static rheology, assessed through steady-state shear testing under specified stress or strain conditions, provides the changes in system viscosity and shear stress as shear rates vary. This method aids in understanding the structural organization and interactions among constituents within an emulsion (Wang et al., 2023).

**Fig. 2:**
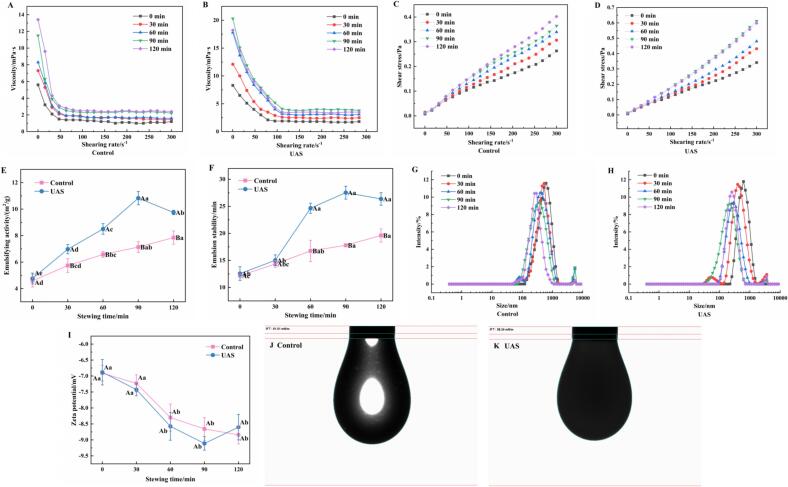
The changes of emulsifying properties of chicken soup of different treatment method: A&B: viscosity, C&D: shear stress, E: emulsifying activity, F: emulsion stability, G&H: particle size, I: zeta potential, J&K: surface tension. Note: Error bars represent standard errors obtained from triplicate sample analysis. Different lowercase letters indicate significant differences (*P* < 0.05) between stewing times, different capital letters indicate significant difference (*P* < 0.05) between the control group and UAS group.

Fig. 2A and Fig. 2B demonstrate the effect of UAS on the viscosity of chicken soup. The viscosity of the control group in the chicken soup displayed an increasing trend as the stewing time progressed. The viscosity of chicken soup is an important indicator of its stability. Stokes' law indicates that the viscosity of chicken soup significantly affects the rate of droplet sedimentation or flotation, with increased viscosity resulting in slower rates. This phenomenon was observed to contribute to the enhanced stability of the chicken soup (Li, Zhao, et al., 2020). At the same stewing time and shear rate, the viscosity observed in the control group of the chicken soup was lower compared to that of the UAS group. An increase in the viscosity of the chicken soup was observed with prolonged UAS, reaching higher values at 90 and 120 min of UAS. The observed increase in viscosity can be attributed to the presence of more complex substances in the UAS group following ultrasonic treatment, such as protein molecules and lipid particles, which interacted to produce greater viscosity (Qi et al., 2023).

Fig. 2C and Fig. 2D illustrate the effect of UAS on the shear stress of chicken soup. As shown in the figure, chicken soup was a non-Newtonian fluid and the shear stress increased with the shear rate. At the same shear rate, the shear stress in the UAS-treated samples surpassed that of the control samples. Table. S1 presents the related parameters of power law curve of shear stress. The correlation between shear stress and shear rate in chicken soup can be described using the power law model. The measurement coefficient R^2^ ranged from 0.97 to 0.99, indicating that the flow curves of the chicken soup were well aligned with the power law model. The coefficient of consistency K reflected the viscosity of the fluid; a higher value of K corresponds to an increased viscosity of the fluid. The K value of all groups increased as the stewing time extended, reaching 0.98375 after 90 min of UAS. This trend was consistent with that observed for viscosity. The flow behavior index (n) reflected the rheological characteristics of the fluid. When the fluid approached Newtonian behavior, the n value was close to 1 (Ribes et al., 2022). All tested groups showed n values less than 1, indicating their pseudo-plastic behavior. The n value of the control samples was lower than that of the UAS samples, indicating a more pronounced shear thinning effect in the control group. The higher n value in the UAS group suggests improved flow properties due to better nutrient migration and distribution, leading to a smoother texture and more uniform consistency, thus enhancing fluid stability compared to the control group.

#### 3.2.2 Emulsifying activity and emulsion stability of chicken soup of different treatment method

Emulsifying activity describes the interactions between proteins or between proteins and fats, while emulsion stability pertains to the maintenance of droplet stability within the emulsion (Wu et al., 2024).

The emulsifying activity in the control sample of chicken soup (4.59–7.85 m^2^/g) continuously increased with increased stewing time, as shown in Fig. 2E. The enhanced emulsifying activity could be attributed to an increase in hydrophobicity, and a potential reduction in particle size. The reduction in particle size led to an expanded protein surface area, while the rise in hydrophobicity facilitated greater exposure of hydrophobic groups for interaction with the oil phase (O'sullivan et al., 2016). Additionally, the unfolding of proteins not only exposed hydrophobic regions, but also hydrophilic regions, enhancing the capacity for better interaction with water and other proteins (Wu et al., 2024), thus elevating the emulsifying activity of the chicken soup. Notably, the emulsifying activity of the UAS sample significantly surpassed that of the control sample (*P* < 0.05), with the UAS group of chicken soup showing a continuous rise (4.76–10.83 m^2^/g) from 0 to 90 min of UAS and a decrease (9.75 m^2^/g) at 120 min of UAS. This may be due to the higher protein content in the UAS sample enhancing the interactions between available proteins, lipids and water, thereby increasing the emulsifying ability, but excessive UAS can reduce the emulsifying activity of chicken soup. As shown in Fig. 2F, the emulsion stability of the control group were gradually enhanced with the increased stewing time (*P* < 0.05). This may be ascribed to the adsorption of proteins at the oil-water interfaces during the processes of heating and stewing (Guan et al., 2023). A notable increase (*P* < 0.05) in emulsion stability was observed following the processing of the soup samples using UAS. The samples treated for 90 and 120 min exhibited higher stability. Two possible explanations are suggested. Firstly, the increased release of water-soluble proteins in the UAS group during the stewing process facilitated interactions with lipids, leading to the formation of stable micro-nano particles (MNPs). Secondly, UAS reduced the size of MNPs particles, increased the contact area between MNPs and the intermolecular force, and contributed to a more effective emulsification process within the chicken soup system.

#### 3.2.3 Particle size and zeta potential of chicken soup of different treatment method

Particle size is an important feature of MNPs, which directly affects the surface properties of MNPs and ultimately shapes the physicochemical and structural characteristics of MNPs (Wu et al., 2024). Protein surface charge density quantified by Zeta potential can be used as an indicator of potential stability in emulsion systems. In general, the higher the absolute Zeta potential of the sample solution, the better the stability (Li, Zhao, et al., 2020).

The changes in particle size in chicken soup over stewing time are depicted in Fig. 2G and Fig. 2H. The particle size of control group was significantly decreased as the stewing was prolonged (*P* < 0.05). Qian et al. (2019) also observed a notable reduction in the average particle size of traditional chicken soup with the extension of the stewing time (*P* < 0.05). This reduction in particle size may be owing to the migration of proteins and lipids from the meat into the soup as the stew progresses, leading to the formation of a stable emulsion under sustained heating, which reduces particle size. Based on DLVO theory and Stokes' law, it is reasonable to consider that smaller droplets require greater kinetic energy to merge with another droplet; thus, smaller droplets are more stable (Miyagawa et al., 2015). For the UAS group, the particle size of MNPs obtained after 90 min of UAS was the smallest. This effect may result from the combined action of UAS induced cavitation and acoustic flow, which enhanced both the rate and intensity of collisions among MNPs in the chicken soup. This disrupted intermolecular hydrophobic interactions among particles, thereby reducing the size of nutrient aggregates, such as proteins and lipids (Jambrak et al., 2014). A narrower range of droplet size distribution was also observed in the UAS samples, indicating that higher homogeneity and increased stability of the emulsion were achieved after ultrasonic treatment (Yao et al., 2021).

The effects of UAS on Zeta potential of chicken soup are depicted in Fig. 2I. As stewing time was extended, the absolute values of the Zeta potential in the control groups increased significantly, reaching a maximum value (8.84 mV) at the end of stewing. The increase was ascribed to the enhanced solubility of proteins in the chicken soup as the stewing was extended, resulting in raised absolute values of the Zeta potential (Guan et al., 2023). For the UAS groups, the absolute value of Zeta potential initially increased and then decreased, with the highest value (9.11 mV) observed at 90 min of UAS. The increase in absolute Zeta potential values following ultrasonic treatment can likely be attributed to cavitation effects, microstreaming, and turbulent forces generated by ultrasound, which mechanically fragmented proteins into smaller particles (Li et al., 2023).

#### 3.2.4 Surface tension of chicken soup of different treatment method

Surface tension is defined as the molecular force acting at the interface between two immiscible media. This tension arises from intermolecular attractive forces, also referred to as surface energy, between the two fluid interface (Ilyas et al., 2020).

Fig. 2J and Fig. 2K displays the surface tension of the control group and UAS group in chicken soup, respectively. The surface tension was determined at 61.01 mN/m in the control group and 58.24 mN/m in the 90-min UAS group. Protein molecules have a tendency to adsorb at interfaces, thereby reducing aqueous surface tension as a result of their surface-active characteristics (Xiao et al., 2020). The UAS groups showed reduced surface tension in comparison to the control group. This may be due to the higher protein content in the UAS soup, where the polar and non-polar functional groups of these protein molecules mixed, exhibiting strong amphiphilicity and leading to their adsorption at the interface, which resulted in a decrease in surface tension. Additionally, the adsorption of spherical protein particles at the oil-water interface reduced interfacial tension, thereby enhancing the stability of the chicken soup.

#### 3.2.5 Appearance and microscopic observation of chicken soup under optical microscope

When chicken soup is stewing, a variety of physical and chemical reactions occur. The chicken soup contains nutrients that have migrated from the chicken, along with new compounds produced by the interactions of various nutrients. These compounds, due to intermolecular interactions, lead to the formation of a substantial quantity of self-assembled particles, which are micro-nano particles (MNPs) (Zhu et al., 2021).

**Fig. 3:**
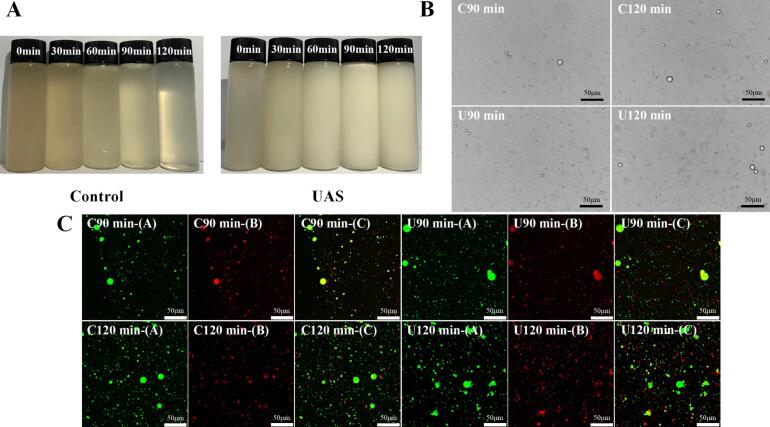
The changes of the general appearance and particle characteristics of chicken soup of different treatment method: A: general appearance, B: MNPs topography of chicken soup under optical microscope, C: MNPs topography of chicken soup under laser scanning confocal microscope. Note: B: Magnification = 400×, scale bar = 50 μm. C: Magnification = 400×, scale bar = 50 μm. (A), (B) and (C) represents triglyceride diagram, protein diagram, and overlap diagram, respectively.

To investigate the impact of UAS on the morphology of chicken soup, the appearance of chicken soup was observed. As shown in Fig. 3A, the control group became increasingly clear as the stewing time increased. When treated with 90 min of UAS, the general appearance of chicken soup changed from transparent to milky white with a uniform texture. After 120 min of UAS, the brightness of the chicken soup decreased, suggesting a reduction in the emulsion stability of the soup. Therefore, UAS could effectively improve the homogeneity and promote the stability of the chicken soup.

Fig. 3B is an optical microscope image of MNPs in chicken soup. As the stewing process progressed for the initial 30 min, lipids, proteins and sugars from the chicken nuggets gradually dissolved into the soup, resulting in the formation of secondary bonds among the molecular components. These interactions gave rise to the development of spherical particles of varying sizes, ultimately leading to the formation of MNPs. Notably, from 30 min onward, the number of particles in the chicken soup exhibited a continuous increase, with nutrient accumulation ensuing as a result of molecular interactions, fostering the self-assembly of complex particles. As depicted in Fig. 3B, between 90 and 120 min, the MNPs in the soup tended to stabilize. By 120 min, the migration of both total lipids and sugars in the soups had peaked, contributing to the formation of MNPs with enhanced stability. The MNPs in both sample groups were spherical. Compared to the control group, the MNPs in the UAS samples were smaller in diameter and size, more uniform in size and more densely arranged. However, larger MNPs occurred due to a decrease in the stability of the chicken soup induced by 120 min of UAS. Therefore, appropriate UAS could effectively enhance the homogeneity of MNPs in chicken soup and promote the stability of the system.

#### 3.2.6 Microstructure observation of chicken soup under laser scanning confocal microscope

Compared to traditional optical microscope, a laser scanning confocal microscope (LSCM) utilizing a laser as a light source. It employs the principle and device of confocal focusing to scan samples and collect signals, subsequently reconstructing 2D and 3D images of the samples through computer processing (Wu et al., 2024).

Proteins (red) and triglyceride (TG) (green) in both soups with stewing time of 90 and 120 min were observed using the Laser Scanning Confocal Microscope (LSCM). Figs. 3C(A) and 3C(B) illustrate the distributions of different components within MNPs. In the control chicken soup, TG and proteins with large diameters were clearly visible, likely due to the aggregation of tiny TG and protein particles. Conversely, the UAS group exhibited small and uniform particles. For both the 90-min and 120-min UAS groups, the smaller and more evenly distributed TG and protein particles were noted, demonstrating the effect of UAS. In Fig. 3C(C), when the fluorescent-stained pictures of proteins and TG were overlapped, it was evident that TG were dispersed as spherical particles of various sizes, while proteins appeared in a cyclic form. The TG were centrally located within the MNPs, with proteins distributed on the periphery. In the 90-min and 120-min UAS groups, the MNPs were smaller in size, larger in quantity and more densely arranged overall compared to the control groups. This finding aligned with the results of the particle size identification. The mechanism of MNPs formation in chicken soup is similar to that in milk. Lopez and Ménard (2011) observed that these particles are oil-in-water colloidal structures, with TG, a crucial component of fat globules, positioned at the center of MNPs and encased by a protein membrane.

### 3.3 Effect of UAS on flavor compounds of chicken soup

#### 3.3.1 GC-IMS topographic plots of chicken soup of different treatment method

GC-IMS is an untargeted analytical methodology employed for the identification of VOCs using retention time and drift time (Wang, Huang, Wang, et al., 2024). and has the advantages of rapid detection, clear results, and portability.

**Fig. 4:**
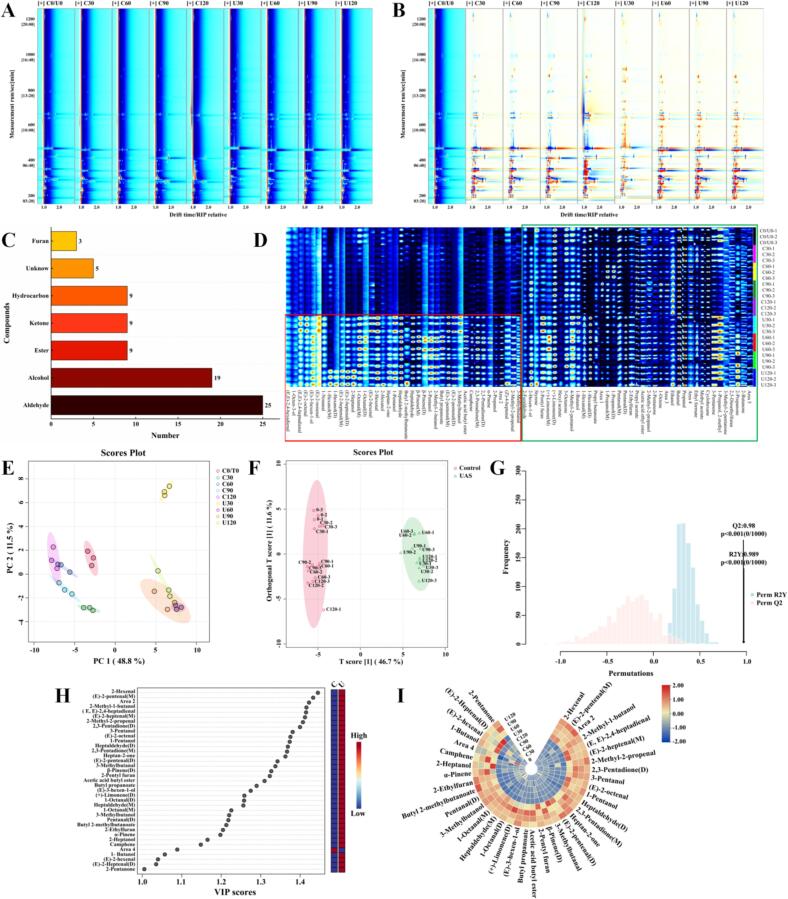
The changes of the volatile compounds of chicken soup of different treatment method: A: topographic plot, B: difference comparison plot, C: categories number plot, D: fingerprints plot, E: PCA plot, F: OPLS-DA plot, G: permutation test based on OPLS-DA analysis, H: VIP scores plots based on OPLS-DA analysis, I: heat map based on VIP > 1. Notes: A-B: Each point around the RIP peak represents a different VOC; the color indicates the concentration of the substance: white for lower concentrations, red for higher concentrations, with darker colors indicating greater concentrations. D: M, D: monomer, dimerization of the same substance, respectively. G: Cross-validation results obtained after 1000 permutation tests. H: Change patterns of volatile flavor compounds with VIP >1 based on OPLS-DA analysis. I: Different colors represent different concentrations. (blue = low, yellow = moderate, red = high). (For interpretation of the references to color in this figure legend, the reader is referred to the web version of this article.)

The VOCs in chicken soup with different treatment methods were described using GC-IMS technology, aiming to understand the changes of VOCs between UAS and control samples. The topographic plot obtained through the GC-IMS analysis was presented in Fig. 4. The red vertical line on the blue background indicates the normalized active ion peak. Each dot to the right of the ion peak (RIP) corresponds to a VOC, with the color gradient indicating the concentration of the substance; higher concentrations are shown in red, while lower concentrations are depicted in white (Yang et al., 2024). Notably, most of the signals detected in the chicken soup samples were in the 200–1000 s retention time range, with a drift time range of 1.0–2.0 s (Fig. 4A). In order to make a clearer comparison of VOC changes between samples, the spectra of C0/T0 were employed as a reference point for comparison with the spectra of other groups. A white background signifies a match with the detected VOCs, whereas red and blue tones denote values that exceed and fall below the reference value, respectively, as illustrated in Fig. 4B. It can be clearly seen that the VOCs content of the UAS group were significantly variation from that of the control group, and the red signal of the UAS chicken soup were the strongest, indicating that the VOCs concentration of the UAS sample were higher than that of the control sample.

#### 3.3.2 Fingerprints of VOCs in chicken soup of different treatment method

To gain deeper insights into the pattern of changes in the VOCs, the GC-IMS database uses migration duration and retention index to accurately locate VOCs, and the results are shown in Table 1 and Fig. 4C. Through GC-IMS analysis, 79 volatile substances were identified, including 25 aldehydes, 19 alcohols, 9 esters, 9 ketones, 9 hydrocarbons, 3 furans and 5 unidentified compounds. It is worth noting that aldehydes and alcohols are the main VOCs in chicken soup. The diversity of VOCs in different treatment groups highlights the complexity of chicken soup aromas, providing a solid basis for future comparative classifications.

**Table 1:** Changes in the content of volatile flavor compounds of in chicken soup.

	Compounds	CAS	Formula	MW	RI	RT	DT
Aldehyde							
1	2-Furaldehyde	C98011	C5H4O2	96.1	1508.3	1229.115	1.06072
2	(E,E)-2,4-hexadienal	C142836	C6H8O	96.1	1506.4	1224.481	1.09461
3	(E, E)-2,4-heptadienal	C4313035	C7H10O	110.2	1493	1192.334	1.16616
4	(E)-2-octenal	C2548870	C8H14O	126.2	1445.9	1085.813	1.33998
5	(E)-2-nonenal	C18829566	C9H16O	140.2	1497.2	1202.285	1.40478
6	1-Nonanal	C124196	C9H18O	142.2	1409.3	1009.603	1.47734
7	(E)-2-heptenal(M)	C18829555	C7H12O	112.2	1331.3	864.639	1.26
8	(E)-2-heptenal(D)	C18829555	C7H12O	112.2	1330.2	862.809	1.67326
9	1-Octanal(M)	C124130	C8H16O	128.2	1297.1	807.834	1.82173
10	1-Octanal(D)	C124130	C8H16O	128.2	1296.4	806.741	1.40437
11	(E)-2-hexenal	C6728263	C6H10O	98.1	1259.5	748.693	1.50955
12	2-Hexenal	C505577	C6H10O	98.1	1230.4	705.727	1.1825
13	Heptaldehyde(M)	C111717	C7H14O	114.2	1194.7	656.354	1.33154
14	Heptaldehyde(D)	C111717	C7H14O	114.2	1195.9	657.925	1.69602
15	(E)-2-pentenal(M)	C1576870	C5H8O	84.1	1146.1	564.117	1.10705
16	(E)-2-pentenal(D)	C1576870	C5H8O	84.1	1146.1	564.117	1.36025
17	1-Hexanal(M)	C66251	C6H12O	100.2	1101.1	488.971	1.26164
18	1-Hexanal(D)	C66251	C6H12O	100.2	1098.7	485.22	1.55683
19	Pentanal(M)	C110623	C5H10O	86.1	999.4	366.88	1.17952
20	Pentanal(D)	C110623	C5H10O	86.1	1000.6	368.137	1.42503
21	Propanal	C123386	C3H6O	58.1	832.2	261.164	1.14162
22	Butanal	C123728	C4H8O	72.1	838.9	264.746	1.11479
23	(Z)-4-heptenal	C6728310	C7H12O	112.2	1294.5	803.744	1.16857
24	2-Methyl-2-propenal	C78853	C4H6O	70.1	896.4	297.419	1.20841
25	3-Methylbutanal	C590863	C5H10O	86.1	866.3	279.864	1.16478

Alcohol							
1	1-Octen-3-ol	C3391864	C8H16O	128.2	1505.9	1223.175	1.16123
2	(E)-3-hexen-1-ol	C928972	C6H12O	100.2	1423.9	1039.32	1.26698
3	1-Hexanol(M)	C111273	C6H14O	102.2	1369.6	932.986	1.32862
4	1-Hexanol(D)	C111273	C6H14O	102.2	1370.9	935.427	1.64627
5	2-Hexen-1-ol	C2305217	C6H12O	100.2	1349.1	895.761	1.17981
6	2-Heptanol	C543497	C7H16O	116.2	1340.2	880.048	1.40707
7	2-Hexanol	C626937	C6H14O	102.2	1229.1	703.843	1.29893
8	1-Pentanol	C71410	C5H12O	88.1	1229.3	704.131	1.51725
9	4-Methyl-2-pentanol	C108112	C6H14O	102.2	1191.7	652.059	1.26112
10	3-Pentanol	C584021	C5H12O	88.1	1173.3	615.044	1.43306
11	2-Methyl-1-butanol	C137326	C5H12O	88.1	1172.5	613.445	1.48328
12	1-Butanol	C71363	C4H10O	74.1	1155.9	582.012	1.1818
13	3-Methylbutanol	C123513	C5H12O	88.1	1146.4	564.611	1.46973
14	1-Propanol(M)	C71238	C3H8O	60.1	1050.5	423.54	1.11328
15	1-Propanol(D)	C71238	C3H8O	60.1	1049.6	422.495	1.24886
16	2-Methyl-2-propanol	C75650	C4H10O	74.1	909.3	305.308	1.32905
17	2-Propanol	C67630	C3H8O	60.1	904	302.067	1.10048
18	1-Propanol, 2-methyl	C78831	C4H10O	74.1	1097.1	482.892	1.34419
19	Ethanol	C64175	C2H6O	46.1	963.6	340.808	1.04162

Ester							
1	Hexyl acetate	C142927	C8H16O2	144.2	1208.2	674.608	1.39818
2	Butyl 2-methylbutanoate	C15706737	C9H18O2	158.2	1192.1	652.779	1.4019
3	Butyl propanoate	C590012	C7H14O2	130.2	1172.8	614.086	1.2822
4	Acetic acid butyl ester	C123864	C6H12O2	116.2	1129.7	535.433	1.21549
5	Propyl butanoate	C105668	C7H14O2	130.2	1082.3	463.179	1.24103
6	Propyl acetate	C109604	C5H10O2	102.1	948.7	330.689	1.13419
7	Acetic acid ethyl ester	C141786	C4H8O2	88.1	928.9	317.648	1.32905
8	Ethyl formate	C109944	C3H6O2	74.1	783.6	236.682	1.09064
9	Methyl acetate	C79209	C3H6O2	74.1	767.7	229.185	1.06114

Ketone							
1	Heptan-2-one	C110430	C7H14O	114.2	1240.1	719.672	1.25397
2	3-Octanone	C106683	C8H16O	128.2	1212	679.782	1.72233
3	2,3-Pentadione(D)	C600146	C5H8O2	100.1	1034.7	405.134	1.21866
4	2,3-Pentadione(M)	C600146	C5H8O2	100.1	1034	404.416	1.29427
5	2-Pentanone	C107879	C5H10O	86.1	921.9	313.228	1.40181
6	3-Pentanone	C96220	C5H10O	86.1	999.1	366.592	1.3465
7	3-Methyl-2-pentanone	C565617	C6H12O	100.2	993.6	362.171	1.48281
8	2-Propanone	C67641	C3H6O	58.1	784.9	237.323	1.12325
9	2-Butanone	C78933	C4H8O	72.1	914.3	308.392	1.24714

Hydrocarbon							
1	Styrene	C100425	C8H8	104.2	1294.8	804.226	1.06108
2	(+)-Limonene(D)	C138863	C10H16	136.2	1213.4	681.786	1.21817
3	(+)-Limonene(M)	C138863	C10H16	136.2	1212.4	680.411	1.29254
4	β-Pinene(D)	C127913	C10H16	136.2	1173.2	614.826	1.21771
5	β-Pinene(M)	C127913	C10H16	136.2	1173.7	615.765	1.70806
6	Camphene	C79925	C10H16	136.2	1071.9	449.878	1.21704
7	α-Pinene	C80568	C10H16	136.2	1035.6	406.212	1.66431
8	1-Octene	C111660	C8H16	112.2	896.6	297.588	1.66133
9	Cyclohexane	C110827	C6H12	84.2	754.1	222.948	1.23018

Furan							
1	2-Pentyl furan	C3777693	C9H14O	138.2	1261	750.954	1.25397
2	2-Ethylfuran	C3208160	C6H8O	96.1	994.4	362.749	1.28715
3	2,5-Dimethylfuran	C625865	C6H8O	96.1	909.1	305.17	1.06007

Unknow							
1	Area 1	–	–	–	1068.5	445.628	1.7996
2	Area 2	–	–	–	898.6	298.767	1.27802
3	Area 3	–	–	–	921.6	313.024	1.86631
4	Area 4	–	–	–	832.5	261.343	1.05736
5	Area 5	–	–	–	926.4	316.035	1.28194

Note: M, D: monomer, dimerization of the same substance, respectively.

GC-IMS fingerprints were accurately analyzed the variations in VOCs in chicken soup using the plug-in Gallery Plot and compared the signal strength of each VOC separately. The color brightness serves as an indicator of the concentration of VOC, the brighter the color represent the VOC is in higher level (Li et al., 2024). Right area denotes the characteristic VOCs of both the control group and UAS group, the VOCs in this area with high contents mainly include 2-pentyl furan, 2-hexen-1-ol, 3-octanone, 4-methyl-2-pentanol, 1-butanol, 1-hexanal(M), 1-propanol(M), pentanal(M), propyl acetate, butanal, propanal, etc. As showen in Fig. 4D, it can be observed that the concentration of VOCs were positively correlated with the stewing time. Strecker degradation and accelerated fat oxidation, caused by high-temperature stewing, result in the creation of more VOCs, enhancing their aroma (Wang, Huang, Yao, et al., 2024). Lower-left area in Fig. 4D denotes the characteristic VOCs of the UAS group, mainly including 2-hexenal, (E)-2-pentenal(M), 2-methyl-1-butanol, (E, E)-2,4-heptadienal, (E)-2-heptenal(M), 2-methyl-2-propenal, 2,3-pentadione(D), 3-pentanol, (E)-2-octenal, etc. Most of these compounds were only found in the UAS group. The cavitation effect of ultrasound promoted the oxidative degradation of amino acids and fats in the chicken, thus significantly affecting the concentration of VOCs (*P* < 0.05) (Wang, Xu, Wang, et al., 2024).

#### 3.3.3 Multivariate analysis of VOCs in chicken soup of different treatment method

In Fig. 4E the PCA score plot is presented, showing a distinct difference between the chicken soups from the UAS group and the control group. The two primary PCA components accounted for 48.8 % and 11.5 % of the distribution, respectively. In total, two separate areas were recognized in the PCA analysis, highlighting clear differences in the VOCs of the UAS and control group chicken soups. Subsequently, OPLS-DA was used to demonstrate clearer clustering, as shown in Fig. 4F. In the two different treatment groups, the samples showed obvious separation trends. The parameters of the OPLS-DA model, R^2^X = 0.467, R^2^Y = 0.954, and Q^2^ = 0.947, suggest that the model is effective. The OPLS-DA model's reliability was confirmed by performing cross-validation with 1000 permutation tests, as shown in Fig. 4G. The intercepts for R^2^ and Q^2^ were 0.980 and 0.989, respectively, which further confirms the model's ability to predict and can be used for subsequent analysis.

VIP are fundamental measures for assessing the importance of variables in differentiating between groups. Ingredients with a VIP score of more than 1 are considered significant contributors, and this threshold is used as a benchmark for identifying key flavor ingredients (Miao et al., 2024). In the chicken soup of different treatment, 37 key VOCs with VIP scores higher than 1 were identified, as shown in Fig. 4H. These included 15 aldehydes, 7 alcohols, 4 ketones, 4 hydrocarbons, 3 esters, 2 furans and 2 unknown substances. These compounds served as differential markers, distinguishing the chicken soup prepared by different treatment methods. In order to study the effects of different treatments on marker VOCs in chicken soup, heat maps were drawn based on the peak intensity of these markers' VOCs. As shown in Fig. 4I, the z-scores represents the peak area of VOCs in the sample. The concentration of UAS group was higher than that in control group, indicating higher VOCs abundance in UAS chicken soup, which was consistent with fingerprint analysis results. The results suggest that ultrasound treatment can markedly intensify the aroma of chicken soup.

### 3.4 Effect of UAS on electronic tongue of chicken soup

**Fig. 5:**
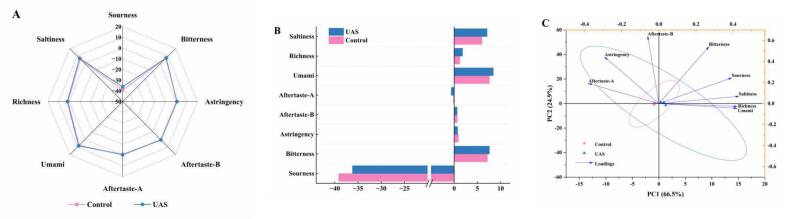
The changes of sensory evaluation analysis of chicken soup of different treatment method: A: radar chart of the five sensory attributes: color, texture, oily, taste, and aroma, B: overall acceptability score. The same letter indicates no significant difference (*P* ≥ 0.05), while different letters indicate a significant difference (P < 0.05).

The electronic tongue technology has the advantages of simple operation, rapidity, analytical efficiency, and the ability to objectively evaluate the taste characteristics of samples (Chen et al., 2023), and the radargram of each of its sensors can reflect the intensity of the corresponding flavor. Thus, the taste of UAS group and control group was evaluated using an electronic tongue, with the findings shown in Fig. 5A and B. It can be observed that each chicken soup sample showed the highest response values to the umami and saltiness sensors, with the UAS group showing higher values than the control group. This may be attributed to the higher concentrations of umami compounds (taste-active amino acids and nucleotides) (Yue et al., 2024). Additionally, most taste parameters on the diagram overlap, indicating that the taste profiles of all samples exhibited a relatively similar taste profile. PCA is a widely used statistical method for visualizing and quantifying differences between samples. In this study, PCA was employed to model and differentiate between chicken soup of different treatment method. The combined interpretation rates of total variance for PC1 (66.5 %) and PC2 (24.9 %) reached 91.4 %, indicating that the extracted principal component features encapsulate a significant portion of the information presented in the original sample data (Peng et al., 2023). As shown in the Fig. 5C, the UAS group and the control group are located in different quadrants, indicating a distinct separation between the different treatment. Moreover, PC1 exhibited positive correlations with umami, richness, saltiness, sourness and bitterness, while displaying negative correlations with astringency, aftertaste-B and aftertaste-A. However, the arrows' lengths were comparable, suggesting that each taste attribute contributed equally to the overall taste profile of the chicken soup. Consequently, the electronic tongue analysis indicates that UAS has a certain promoting effect on the taste of the chicken soup.

### 3.5 Effect of UAS on sensory evaluation of chicken soup

**Fig. 6:**
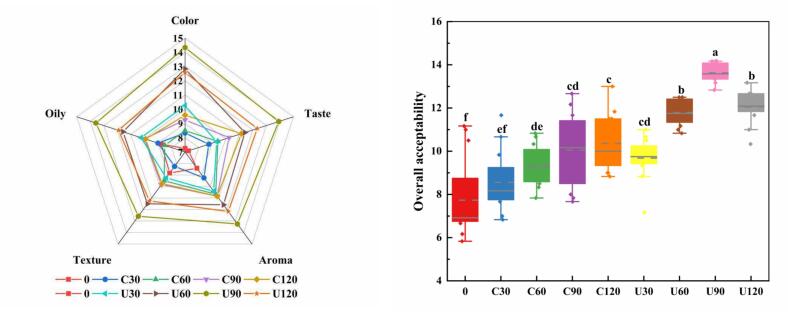
The changes of electronic tongue analysis of chicken soup of different treatment method: A: radar chart, B: classification bar chart, C: biplot analysis chart.

To better understand the effects of UAS on the changes in taste and aroma of soup, the sensory evaluation of chicken soup was carried out. As presented in Fig. 6, the color, texture, oily, taste, aroma and overall acceptability generally showed an increasing trend with the increase of stewing time. This conclusion has also been validated by the study of Guan et al. (2023). UAS significantly enhanced (*P* < 0.05) all sensory indicators of the chicken soup. After UAS, the increase in color, texture, oily may be attributed to the cavitation effect of ultrasound, which breaks down fat particles into smaller droplets, thereby enhancing emulsification, improving texture, and enhancing the color and oily of the soup (O'sullivan et al., 2016). The increase in taste may be attributed to the significant release and dispersion of various nutrients such as proteins, fats and sugars. UAS promoted better dissolution and interaction of these components, resulting in a richer and more complex taste (Zhu et al., 2021). The increase in aroma may be due to the enhanced release of VOCs, which is promoted by both ultrasonic treatment and heat treatment, resulting in a more pronounced and complex aroma profile (Qi et al., 2023). However, after 120 min of UAS, all sensory indicators showed a decline, which may be related to the gradual reduction of dissolved proteins in the soup during the late period of UAS. Notably, the overall acceptability of the UAS group exhibited a statistically significant enhancement over the control group (*P* < 0.05). This data suggests that the UAS could significantly enhance the sensory characteristics of chicken soup.

### 3.6 Correlation analysis between nutritional content, emulsifying properties and volatile compounds in chicken soup

**Fig. 7:**
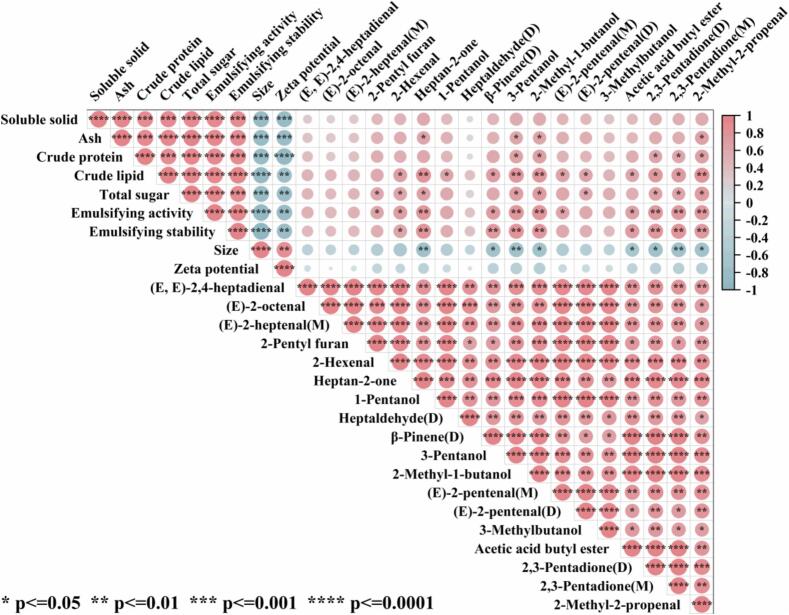
Correlation analysis between nutritional content, emulsifying properties and volatile compounds in chicken soup.

The correlation between VOCs and nutritional and emulsifying indicators in chicken soup were investigated using Pearson correlation analysis (Fig. 7). In this study, 18 major differential VOCs (VIP >1.3) in the chicken soup treated by different methods were used for correlation analysis. As shown in Fig. 7, all the content of nutritional (soluble solids, total sugar, crude protein, crude lipids and ash) were significantly and positively correlated with emulsifying activity, emulsifying stability (*P* < 0.001), while there was a significant negative correlation with size and Zeta potential (*P* < 0.01). The results show that the application of UAS technology can continuously release the nutrients in the chicken into the soup, thus enriching the nutrients and improving the emulsifying properties of the soup. The results indicate that the UAS process allowed the nutrients from the chicken to consistently dissolve into the soup, thereby enhancing the nutrient levels and improving the emulsifying characteristics of the chicken soup. Negative Zeta potential values were inversely correlated with protein content, emulsifying activity and emulsion stability, indicating that long stewing is conducive to the development of many protein structures. This causes the amino acids to migrate outward and increases the negatively charged amino acids in chicken soup (Wu et al., 2024). Lipid oxidation is a key pathway for the formation of VOCs during product processing (Yang et al., 2024). In this study, there was a statistically significant positive correlation between lipid content and VOCs such as 2-hexenal, heptan-2-one, 3-pentanol, 2-methyl-1-butanol, (E)-2-pentenal(M), etc. (*P* < 0.05), highlighting the key role of lipid oxidation in the formation of these VOCs. In addition, furan is an important VOC produced during heat treatment of meat products and is a volatile group derived from the Maillard reaction (Yang et al., 2024). The contents of total sugar and crude protein were significantly positively correlated with 2-pentyl furan (*P* < 0.05), indicating that they contributed significantly to the production of these VOCs.

## 4 Conclusions

In summary, this study demonstrates that ultrasonic-assisted stewing (UAS) of chicken soup is an effective strategy for improving the nutritional value, emulsifying properties, aroma intensity, and sensory attributes of the soup. The UAS group showed higher nutrient contents, rheological properties and emulsification performance compared to the control group. These improvements were attributed to the MNPs were smaller and more uniformly distributed of the UAS-treated chicken soup. In terms of flavor characteristics, UAS significantly improved the aroma intensity of chicken soup, with the UAS group exhibiting a richer variety of flavor compounds. Additionally, sensory evaluation and electronic tongue analysis revealed that the UAS group had better taste attributes than the control group. Treatment for 90 min with UAS markedly optimized the quality of the chicken soup. These benefits were achieved through the breakdown of raw material tissues and protein fibers through the shear, cavitation, and homogenization effects of UAS. This study has practical applications in improving the quality of chicken soup and holds great importance for the industrial production of chicken soup.

## CRediT authorship contribution statement

**Ziyan Yue:** Writing – original draft, Methodology, Investigation, Data curation. **Qiuyu Yu:** Software, Investigation. **Yuchen Qin:** Visualization, Investigation. **Yuchun He:** Visualization, Resources. **Jiali Liu:** Software, Resources. **Yingchun Zhu:** Writing – review & editing, Validation, Methodology, Formal analysis.

## Declaration of competing interest

The authors declare that they have no known competing financial interests or personal relationships that could have appeared to influence the work reported in this paper.

## Data Availability

Data will be made available on request.

## Appendix A Supplementary data

Supplementary material Image 1
