# Supplementary material for: Enhancement of quality induced by ultrasonic-assisted stewing improved the nutritional concentration, emulsifying property, and flavor characteristic of the chicken soup
Source: Food Chem X. 2025 Jan 15;25:102184. doi: 10.1016/j.fochx.2025.102184 (PMC11788733; doi:10.1016/j.fochx.2025.102184)
Supplement: Supplementary file 1 — Supplementary material [file mmc1.docx]

**Supplementary material**

Enhancement of quality induced by ultrasonic-assisted stewing improved the nutritional concentration, emulsifying property, and favor characteristic of the chicken soup

Ziyan Yue, Qiuyu Yu, Yuchen Qin, Yuchun He, Jiali Liu, Yingchun Zhu*

College of Food Science and Engineering, Shanxi Agricultural University, Taigu 030801, China

*****Corresponding authors: Yingchun Zhu

Tel.: +86 18503449054

E-mail addresses: yingchun0417@163.com (Y.C. Zhu).

**Table S1**

Related parameters of power law curve of shear stress

| Treatment | K (viscosity coefficient) | n (fluid index) | R^2^ |
| --- | --- | --- | --- |
| C 0 min | 0.77974 | 0.40514 | 0.99419 |
| C 30 min | 0.8232 | 0.41084 | 0.99851 |
| C 60 min | 0.8547 | 0.41337 | 0.99905 |
| C 90 min | 0.88689 | 0.41943 | 0.99236 |
| C 120 min | 0.90378 | 0.42080 | 0.99814 |
| T 0 min | 0.82388 | 0.40654 | 0.99639 |
| T 30 min | 0.86899 | 0.41469 | 0.97365 |
| T 60 min | 0.90529 | 0.41825 | 0.98899 |
| T 90 min | 0.98375 | 0.42941 | 0.99435 |
| T 120 min | 0.95899 | 0.42225 | 0.99119 |
